# Antiproliferative property of hexadecyloxypropyl 9-[2-(phosphonomethoxy) ethyl] guanine (HDP-PMEG) for unwanted ocular proliferation

**Published:** 2011-03-02

**Authors:** Jiangping Hou, Yuli Li, Zhonglou Zhou, Nadejda Valiaeva, James R. Beadle, Karl Hostetler, William R. Freeman, Dan-Ning Hu, Hao Chen, Lingyun Cheng

**Affiliations:** 1 Institute of Ocular Pharmacology, School of Ophthalmology and Optometry, Wenzhou Medical College, Wenzhou, Zhejiang, China; 2 Department of Medicine, University of California, San Diego and the San Diego Veterans Medical Research Foundation, La Jolla, CA; 3 Jacobs Retina Center at Shiley Eye Center, Department of Ophthalmology, University of California San Diego, La Jolla, CA; 4 Ophthalmology, The New York Eye and Ear Infirmary, New York, NY

## Abstract

**Purpose:**

To investigate the safety and inhibitory effects of hexadecyloxypropyl 9-[2-(phosphonomethoxy) ethyl] guanine (HDP-PMEG) on ocular cell proliferation and collagen matrix contraction.

**Methods:**

For the antiproliferation studies, various ocular cell monolayers were exposed to HDP-PMEG, PMEG, 5-fluorouracil (5-FU), and daunorubicin (DNB). For the collagen contraction studies, retinal pigment epithelium (RPE) cells seeded onto type I collagen lattices were exposed for a single 5- or 50-min period to various concentrations of HDP-PMEG or 5-FU. For the cytotoxicity study, trypan blue exclusion tests were performed using a human Müller cell line. Cytotoxicity was determined up to 4 days after treatment.

**Results:**

The proliferation of RPE cells, scleral fibroblasts, vessel endothelial cells, and ocular melanoma cells can all be significantly inhibited by HDP-PMEG. Its inhibitory effects on those cells were uniformly stronger than that of 5-FU. Contraction of the collagen matrix containing RPE cells was significantly inhibited by HDP-PMEG and by 5-FU at concentrations of 20 µM and 2,000 µM, respectively, as compared with controls (p<0.05). The safety profile of HDP-PMEG was significantly better than 5-FU and daunorubicin. The ocular therapeutic index is 1,100 for HDP-PMEG, 17.2 for 5-FU, and 1.25 for daunorubicin.

**Conclusions:**

HDP-PMEG possesses a significant inhibitory effect on the proliferation of RPE, retinal glial cells, scleral fibroblasts, and ocular melanoma cells. HDP-PMEG is also genotoxic and may be used as a single short application for the modulation of unwanted ocular proliferation.

## Introduction

Unwanted ocular proliferation consists of an array of diseases such as proliferative vitreoretinopathy (PVR) and retinal surface proliferation including epiretinal membrane (ERM) and macular pucker. Their pathophysiology involves many aspects of cell biology and studies have suggested that retinal pigment epithelial (RPE) cells and glial cells are the major cell components of retina proliferation [1,2]. In addition, pathological angiogenesis and abnormal vessel growth are involved with these scarring processes [3]. These diseases share a common feature in which the participating cells lose their quiescent nature and trans-differentiate to fast proliferating cells. Inhibition of the proliferation of those cells has been investigated, including the use of the FDA-approved antiproliferative drugs daunorubicin [4] and 5-flourouracil [5]. However, no ideal compound or formulation has been identified for effective clinical treatment because of the potential toxicity or the insufficient evidence for efficacy [4,5].

**Figure 1:**
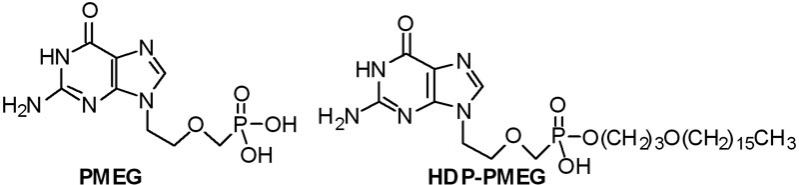
Chemical structure of PMEG and HDP-PMEG.

Acyclic nucleoside phosphonates (ANPs) are nucleotide analogs with significant antiviral, cytostatic and antiproliferative activities [6]. Three ANPs (cidofovir, adefovir, and tenofovir) are FDA-approved as antivirals [7]. Among ANPs with inhibitory effects toward dividing cells, 9-[2-(phosphonomethoxy)ethyl] guanine (PMEG, Figure 1) is one of the most potent [8], the genotoxicity of PMEG being comparable to that of mitomycin C [9], an inhibitor of DNA synthesis which is administered as a single short exposure during anti-glaucoma filtering surgery to prevent fibroblast proliferation at the surgical site [10]. In proliferating cells, PMEG is metabolized to PMEG diphosphate (PMEGpp), a potent inhibitor of replicative human polymerases α, δ, and ε, resulting in cytotoxicity [8]. PMEG is active against leukemia and melanoma in animal tumor models [11], and prodrug derivatives related to PMEG have been synthesized that are effective against lymphoma in dogs [12]. Due to its potent antiproliferative effects, we felt that PMEG may also be a good candidate for local treatment of ocular proliferative diseases. However, the utility of intravitreally administered PMEG is limited by its short intravitreal residence time and toxicity, especially ocular hypotony [13]. Therefore, successful intravitreal application of PMEG and other antiproliferative ANPs may depend on the development of new derivatives with reduced toxicity, enhanced pharmacokinetic properties (i.e., sustained release), and the potential to selectively deliver high concentrations of the active diphosphate to ocular tissues.

To achieve this objective, we focused on the previously reported lipophilic PMEG prodrug, hexadecyloxypropyl-PMEG (HDP-PMEG, Figure 1) [14]. Earlier studies have shown that the antiviral activity of PMEG and other ANPs can be greatly increased by esterification with alkoxyalkyl groups such as hexadecyloxypropyl (HDP) due to increased cell entry of the lipid-modified drug leading to higher intracellular levels of the active diphosphate metabolite [15]. In addition, we showed previously that alkoxyalkyl modified ANPs and nucleoside monophosphates are sparingly soluble in vitreous, and as a result, are slowly released from an intravitreal depot to produce a sustained release effect [16,17]. This study was designed to evaluate the potential of HDP-PMEG for local, slow-release therapy of PVR, single infusion during a vitrectomy, or as a short contact application for preventing excessive fibrosis following anti-glaucoma filtering surgery. To this end, we examined the in vitro antiproliferative activity of HDP-PMEG against six human cell lines, including cell components involved in vitreoretinal proliferation and ocular melanoma. To gain additional evidence of antiproliferative activity, we also assessed the ability of HDP-PMEG to prevent collagen matrix contraction.

## Methods

### Antiproliferative activity assay and cytotoxicity study

HDP-PMEG was synthesized as previously described [14]. The current study was undertaken to test the possibility of an ocular application of HDP-PMEG to treat unwanted intraocular proliferation as well as ocular neoplasm which also involves fast cell proliferation. The antiproliferation activity of HDP-PMEG was tested on six human cell lines: two human retinal cell lines, including pigment epithelium ARPE19 (CRL-2302; ATCC, Manassas, VA) and Müller cells (MIO-M1, a gift of Dr. Gloria Astrid Limb, Ocular Repair and Regeneration Biology Unit, Departments of Cell Biology and Pathology, Institute of Ophthalmology, London, UK) [18]; a human endothelial-like immortalized cell line EA-HY926, derived from the fusion of human umbilical vein endothelial cells (HUVEC) with the lung carcinoma cell line A549 (CRL-2922; ATCC); and three ocular uveal melanoma cell lines, including OCM1 [19], M23 (established in the New York Eye and Ear Infirmary, New York, NY) [20] and SP6.5 (kind gift from Dr. Guy Pelletier, Research Center of Immunology, Quebec, Canada) [21]. The first three cell types are the major cell components involving vitreoretinal proliferation; the last three cell types represent ocular melanoma.

ARPE19 cells were cultured in DMEM/Ham’s F12 (1:1; Gibco, Invitrogen, Paisley, UK) supplemented with 10% FBS (Gibco, Invitrogen) and OCM1 cells were cultured in Nutrient Mixture Ham’s 1640 RPMI medium (Gibco, Invitrogen) supplemented with 10% FBS. M23, SP6.5, EA.HY926 and Müller cells (MIO-M1) were cultured in DMEM (Gibco, Invitrogen) containing 10% FBS. The parallel studies were performed using PMEG and the other two well known antiproliferation agents, 5-fluorouracil (5-FU; Sigma, St. Louis, MO) and daunorubicin (DNB; Sigma). Stock solutions of HDP-PMEG and PMEG were made in DMSO (Sigma) and stock solutions of 5-FU and daunorubicin were made in PBS. All testing concentrations were diluted from the stock solution using cell culture medium supplemented with 2% FBS. The final highest concentration of DMSO was 0.08% which was confirmed not toxic to the cells used in the current study in a separate study (data not shown). Cells cultured without the drug were used as controls. Final concentrations of the testing compounds were 20 μM, 10 μM, 1 μM, 0.1 μM, 0.01 μM and 0.001 μM; 20 µM was the highest possible concentration tested for HDP-PMEG due to its poor solubility, though 50 μM and 100 μM concentrations were also tested for the other compounds. The cells were seeded into a 96 well plate with 5,000 cells per well and were incubated at 37 °C in 5% carbon dioxide which will reach near 100% confluence in control wells within the projected 4-day culture. After 4 days, the cultured cells will manifest a contact inhibition effect, which could potentially confound the results of the proliferation study, so day 4 was used as the study end point. After 4 days of incubation with 200 µl test solution, cell viability was measured using Promega MTS (Promega Corporation, Madison, WI) assay in which the conversion of MTS into aqueous, soluble formazan is accomplished by dehydrogenase enzymes found in metabolically active cells. The quantity of formazan product as measured by the amount of 490nm absorbance is directly proportional to the number of living cells in culture. The study was repeated three times and five replicates were used within each study.

For the cytotoxicity study, trypan blue dye (Sigma) exclusion tests were performed using the Müller cell line, which originated from human retina and has a slower replication than ARPE19 or the other cells used in this study, to better represent quiescent retina cells. The Müller cell culture and drug exposure was the same as described in the above MTS assay. The trypan blue staining in the 96-well plate was evaluated after a 4-day exposure to HDP-PMEG, 5-FU, Daunorubicin, or 2% DMEM (control). Five images from 5 different locations in each well were acquired immediately after removing the dye. The total cell count and the trypan blue positive cell count for each well were calculated from the images and expressed as a percentage.

### Antiproliferation study after a single, short-term exposure

Fifty micro liters of either the ARPE19, or Müller cell, or anterior scleral fibroblast [22] suspension (2,000 cells) was added into each well of a 96-well plate. After the cells were well adhered to the plate’s bottom, the cells were then exposed, in sextuplicate, to 200 μl/well of HDP-PMEG, 5-FU, or serum-free DMEM/F12 medium (control) for a period of 5 or 50 min. The 5-min exposure was used as an intended implication for intraoperative use for anti-glaucoma filtering surgery and a 50-min contact was used as an intended implication for infusion during a vitrectomy for PVR. The concentrations used were 20 μM, 6.32 μM, and 2 μM for HDP-PMEG and 20 mM, 6.32 mM, 2 mM, 200 μM, and 20 μM for 5-FU (sigma). After an exposure of 5 or 50 min, the solution containing the test drug was removed and 200 μl fresh culture medium was added back into each well. The cultures were incubated at 37 °C in 5% carbon dioxide for 7 days with the culture medium changed once at day 3. To better assess the genotoxicity of the compounds, fewer cells (2,000) were used to start and a longer observation (7 days) was performed. Cell proliferation was assessed by using the MTS assay as described above.

### Collagen matrix contraction study

Collagen gels were prepared as described by Shawn et al. [23] using collagen type I (BD Biosciences, San Jose, CA) and DMEM/F12 (Gibco, Invitrogen) at a final concentration of 2.0 mg/ml. Freshly prepared collagen solution (500 μl) was added into each well of a 24-well plate and incubated at 37 °C in 5% CO_2_ for 1 h. A 500 µl suspension containing 1×10^5^ ARPE19 cells were added to the collagen matrix. RPE cells were used because the retinal pigment epithelium is the major participating cell in PVR formation [2,23,24]. An overnight incubation allowed for the RPE cells to adhere to the collagen gel, after which 500 μl of the test drug solutions were added and incubated for either 5 min or 50 min. Each drug concentration was tested in triplicate. For HDP-PMEG 20 μM, 6.32 μM, and 2 μM were tested and concentrations of 2 mM, 200 μM, and 20 μM for 5-FU. The medium, DMEM/F12, was used as the control. Four days after drug exposure, the collagen gel was photographed using a digital camera and the gel sizes were quantified using Image-Pro Plus 6.0 software. Each drug concentration was tested in triplicate and each experiment was repeated three times.

### Statistical analysis

One-way ANOVA was used for the analysis of all results. Multiple comparisons among various concentrations and controls were made and the observed significance levels were adjusted using the Bonferroni or Dunnett methods. A p value smaller than 0.05 was considered to be statistically significant. All statistical analysis was performed using SAS software (SAS version 9.2, Cary, NC).

## Results

### Antiproliferation potency

**Figure 2:**
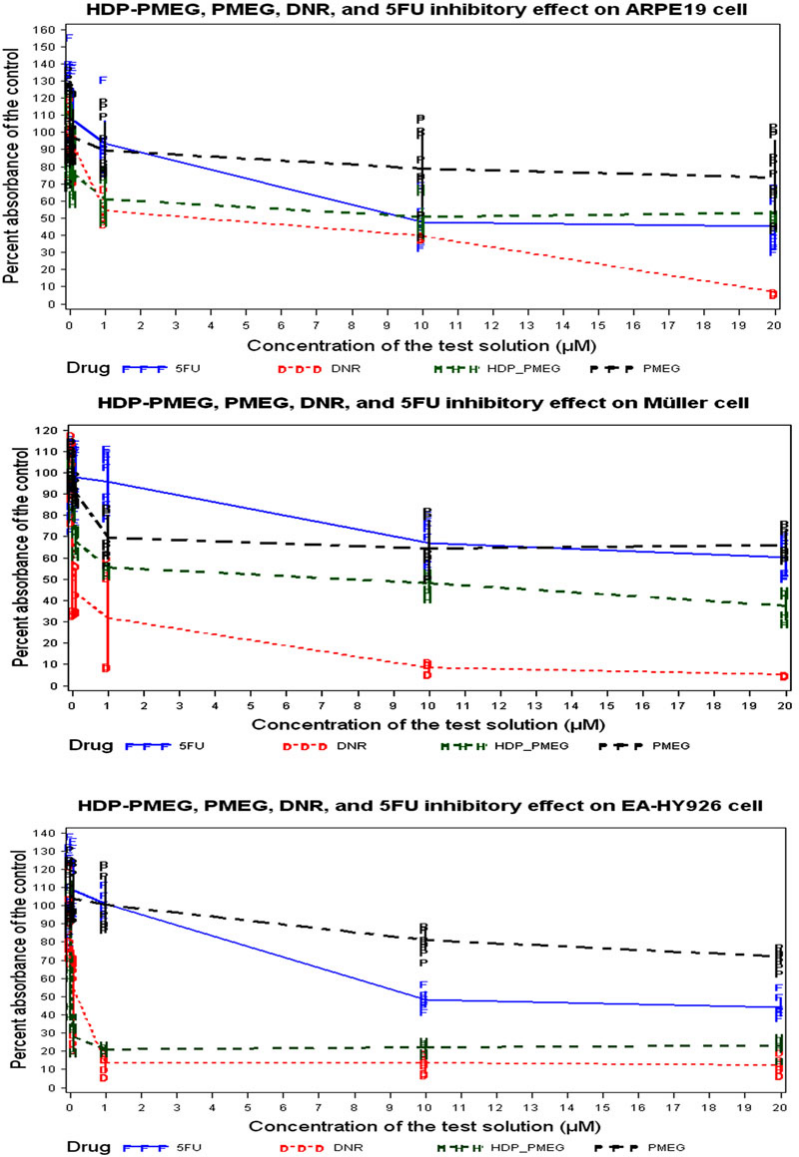
MTS cell proliferation assay. Upper panel: proliferation assay on ARPE19 cell. Center panel: proliferation assay on Muller cell. Bottom panel: proliferation on EA-HY926 endothelium cell.

**Figure 3:**
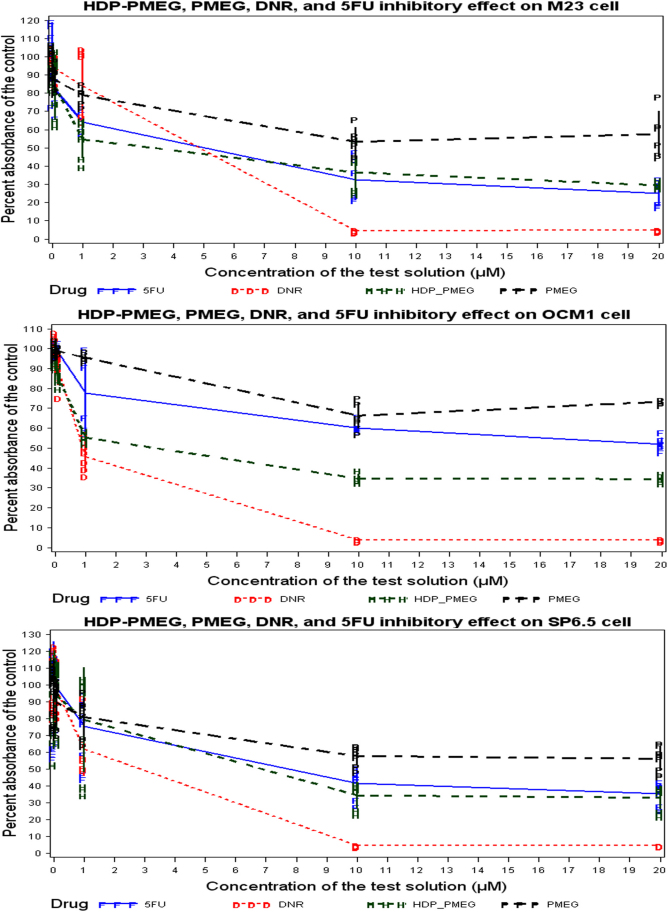
MTS cell proliferation assay to demonstrate the inhibitory effect on proliferation of the three melanoma cell lines: M23 cell (Upper panel), OCM1 cell (Center panel), and SP6.5 cell (Bottom panel). For M23 and SP6.5 cells, HDP-PMEG demonstrated a similar inhibitory effect to 5-FU while HDP-PMEG had a consistent stronger inhibitory effect than 5-FU on the OCM1 cell. Daunorubicin had the strongest inhibitory effect on all the three cell lines.

HDP-PMEG inhibited 40% RPE cell growth at 1 µM while 5-FU achieved similar inhibition at 8 µM. At 1 µM HDP-PMEG had similar inhibitory strength to daunorubicin. For Müller cells, the inhibitory strength of HDP-PMEG was stronger than 5-FU at all tested concentrations and its strength came in between 5-FU and daunorubicin. For the endothelium-like cell line EA-HY926, HDP-PMEG demonstrated a similar inhibitory strength to daunorubicin and was much stronger than 5-FU (Figure 2). For M23 and SP6.5 cells, HDP-PMEG demonstrated a similar inhibitory effect to 5-FU, while HDP-PMEG had a consistently stronger inhibitory effect than 5-FU on OCM1 cells. Daunorubicin had the strongest inhibitory effect on all three melanoma cell lines (Figure 3). Table 1 summarizes the characteristics of the four compounds using the IC_50_ (the concentration needed to inhibit the 50% of the cell proliferation), the minimum effective concentration, which was the lowest concentration showing significant proliferation inhibition as compared to the control, and the mean MTS readings at 1 µM which was in the mid-range of the tested concentrations.

**Table 1:** Antiproliferative compounds and potency profile.

**Compound**	**Cell line**	**Minimum effective concentration tested (μM)**	**IC_50_ (μM)**	**Mean OD value of MTS reading at 1 μM**	p value*
PMEG	ARPE-19	10	>50	1.01	0.0002
5-FU	ARPE-19	10	20	1.05	<0.001
DNR	ARPE-19	1	5	0.7	0.99
HDP-PMEG	ARPE-19	0.1	10	0.71	—
PMEG	EA-HY926	10	50	1.6	<0.001
5-FU	EA-HY926	10	10	1.5	<0.001
DNR	EA-HY926	0.001	0.3	0.21	0.13
HDP-PMEG	EA-HY926	0.01	0.05	0.36	—
PMEG	MIO-M1	1	>50	0.98	0.82
5-FU	MIO-M1	10	>100	1.54	<0.001
DNR	MIO-M1	0.1	0.1	0.5	0.0142
HDP-PMEG	MIO-M1	0.01	10	0.9	—
PMEG	M23	0.1	50	1.51	0.0029
5-FU	M23	0.1	3	1.18	0.57
DNR	M23	1	2	1.38	0.0301
HDP-PMEG	M23	0.1	3	1.04	—
PMEG	OCM1	10	50	2.26	<0.001
5-FU	OCM1	1	20	1.81	0.012
DNR	OCM1	0.1	1	0.93	0.064
HDP-PMEG	OCM1	0.01	3	1.32	—
PMEG	SP6.5	1	50	1.65	0.93
5-FU	SP6.5	1	10	1.55	0.99
DNR	SP6.5	1	2	1.06	0.0682
HDP-PMEG	SP6.5	1	5	1.56	—

IC_50_: drug concentration needed to inhibit cell proliferation by 50%. Minimum effective concentration: minimum concentration tested showing inhibitory action compared with the control. *: p Value derived from comparing the mean OD values at 1 μM on the same cell line using HDP_PMEG as control (Dunnett method).

### Cytotoxicity evaluation

**Figure 4:**
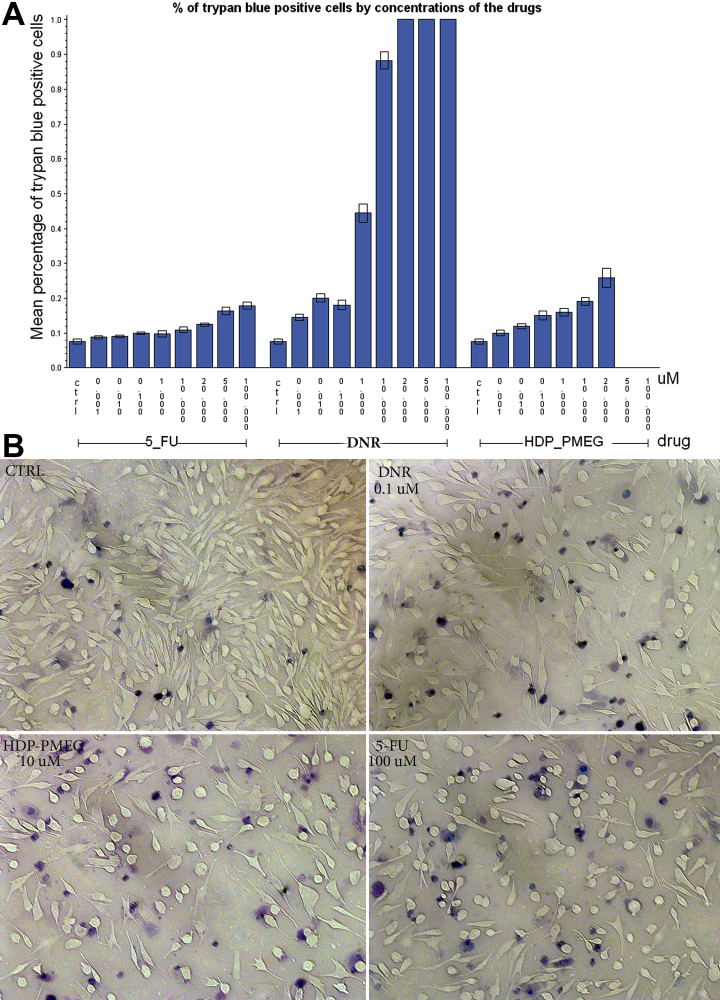
Cytotoxicity assay on Müller cell. **A**: The percentage of trypan blue positive cells (y-axis) was plotted against the concentration of the test compounds used (x-axis). The concentration unit was micro molar and the open box represented a 95% confidence limit. 5-FU at 100 µM that was the highest concentration tested, caused 20% death of the cell culture, which was equivalent to HDP-PMEG at 11 µM or DNR at 0.1 µM. **B**: Exemplary images from the Müller cell cytotoxicity study: the upper left panel showing a counting field from the control in which 31 cells were stained by trypan blue out of a total of 435 cells, yielding a 7% trypan blue positive rate. The rest of the three fields had a trypan blue positive rate of 19% (38/198) for 10 µM HDP-PMEG, 18% (46/251) for 100 µM 5-FU, and 18% (49/274) for 0.1 µM daunorubicin (DNR).

Figure 4A demonstrates the drug cytotoxicity results from the trypan blue dye exclusion assay. All three drugs showed a higher mean percentage of trypan blue positive cells as compared to the controls even at a 0.001 µM concentration (5-FU: 8.8% versus 7.6%, p=0.0353; DNR: 14.6% versus 7.6%, p<0.0001; HDP-PMEG: 10% versus 7.6%, p=0.0176 Adjusted for multiple comparisons with Dunnett). The TC_50_ (the concentration to cause 50% cell death) for HDP-PMEG and 5-FU was not reached in this study and for DNR was 3 µM; however, the TC_20_ was available from the study and it was 11 µM for HDP-PMEG, 100 µM for 5-FU, and 0.1 µM for DNR (Figure 4B). To estimate the ocular therapeutic index (TI=TC_50_/IC_50_) which is defined as the ratio of drug efficacy to the magnitude of its adverse side effects, we used TC_20_ and IC_20_ to calculate the TI. TI was 17.2 for 5-FU, 1,100 for HDP-PMEG, and 1.25 for DNR. A high ocular TI will suggest that the compound might prevent proliferation of cells in vivo without affecting non-dividing cells such as those of the retina.

### Antiproliferation effect from the short contact

**Figure 5:**
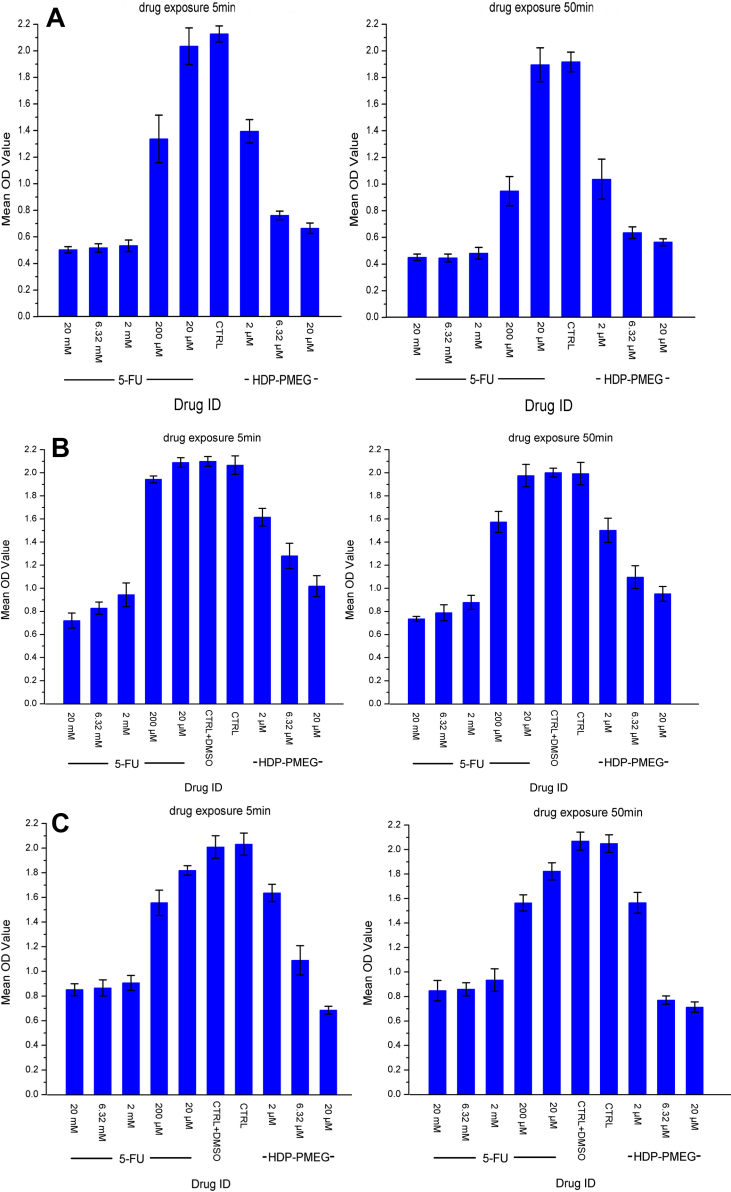
The ARPE19 cell proliferation inhibitory effect from single short-term exposure of HDP-PMEG and 5-FU. **A**: Following a 50-min contact, 2 µM and 6.32 µM HDP-PMEG inhibited cell proliferation by 50% and 75%, respectively, which were equivalent to 200 µM and 2,000 µM of 5-FU. The 5-min exposure also induced significant proliferation inhibition. However, the inhibition was consistently less than that from 50-min exposure cross all concentrations. The mark on the top of each bar represents the standard deviation. **B**: The Müller cell proliferation inhibitory effect from a single short-term exposure of HDP-PMEG and 5-FU. Both 5- and 50-min exposure induced dose dependent inhibition by 5-FU and HDP-PMEG. In a 50-min exposure, 2 µM HDP-PMEG induced a similar growth inhibition as that observed by 200 µM 5-FU. **C**: The anterior scleral fibroblast proliferation inhibitory effect from a single short-term exposure of HDP-PMEG and 5-FU. For the anterior scleral fibroblasts, a 20 µM HDP-PMEG 5-min exposure induced a 65% inhibition while even 20 mM 5-FU did not achieve the similar magnitude growth inhibition.

Drug exposure at both 5 min and 50 min caused significant proliferative inhibition for both 5-FU and HDP-PMEG (Figure 5A-C) on all three tested cell lines. As a whole, the inhibition was significantly more with the 50 min contact (p<0.0001). A 50-min exposure to 2 µM HDP-PMEG induced a 50% inhibition of ARPE19 cells during a 7-day observation. In the same study, a 200 µM 5-FU 50-min exposure achieved a similar percentage of proliferation inhibition. The proliferative inhibition was dose-dependent for HDP-PMEG, while for 5-FU, doses higher than 200 µM caused a similar degree of proliferation inhibition (25% of the control). For anterior scleral fibroblasts, a 20 µM HDP-PMEG 5-min exposure induced a 65% inhibition while even a 20 mM 5-FU did not achieve the similar magnitude growth inhibition (p<0.0001, Figure 5C).

### Collagen matrix contraction study

The collagen matrix contraction study showed a dose-dependent inhibition of collagen matrix contraction for both HDP-PMEG and 5-FU (Table 2). For both HDP-PMEG and 5-FU, a 50-min exposure led to a more significant inhibition than that from the 5-min exposure (p<0.0001). For HDP-PMEG, 6.32 and 20 µM significantly inhibited the collagen matrix contraction and their effects are equivalent to 200 and 2000 µM of 5-FU, respectively (Table 2). Two micromolar HDP-PMEG and 20 µM 5-FU did not show significant inhibition of collagen matrix contraction.

**Table 2:** Collagen matrix contraction following single short-term contact of HDP-PMEG or 5-FU.

**Concentration**	**LS mean area of collagen matrix (mm^2^)**	**p value versus ctrl**	**Concentration**	**LS mean area of collagen matrix (mm^2^)**	**p value versus ctrl**
**HDP-PMEG (5 min)**			**5-FU (5 min)**		
20 µM	53.82	0.026	2000 µM	50.93	<0.0001
6.32 µM	44.63	0.6	200 µM	50.03	<0.0001
2 µM	42.53	0.91	20 µM	35.57	0.31
ctrl	41.96	—	ctrl	31.63	—
**HDP-PMEG (50 min)**			**5-FU (50 min)**		
20 µM	116.51	<0.0001	2000 µM	110.83	<0.0001
6.32 µM	83.93	0.0007	200 µM	82.09	0.0003
2 µM	53.86	0.92	20 µM	56.31	0.63
ctrl	54.64	—	ctrl	52.79	—

ctrl: control; versus: versus; LS mean: least square mean; min: minute. p value was derived from comparing least square mean of each concentration with the least square mean of the control with adjusting for multiple comparisons with Dunnett.

## Discussion

The current study demonstrated that HDP-PMEG possesses a potent antiproliferative property. At present, PVR is still being treated by surgical procedures and pharmacological prevention or early treatment needs to be further explored. 5-FU was the first FDA approved small molecule antimetabolite interfering with DNA and RNA synthesis. Blumenkranz et al. demonstrated effectiveness of 5-FU in in vivo experimental models of PVR [25]. However, the recent clinical trials using 5-FU as a perioperative infusion for the management of PVR did not show significant therapeutic benefit [5,26,27]. Those clinical studies used 200 µg/ml of 5-FU for perioperative infusion [5,26,27]. In the current study, we used 260 µg/ml (2,000 µM) of 5-FU which demonstrated efficacy in both the antiproliferation assay and the collagen matrix contraction assay. The equivalent antiproliferative strength seen with 2,000 µM of 5-FU was achieved by using 20 µM of HDP-PMEG in the collagen contraction study. The antiproliferation assay revealed that the ocular therapeutic index for HDP-PMEG was 64 times better than 5-FU, which indicates a better efficacy and safety profile for HDP-PMEG. The current study also demonstrated better inhibitory potency of HDP-PMEG than 5-FU on blood vessel endothelium (EA-HY926) proliferation, which is a key process for many types of pathological proliferation. Along with HDP-PMEG and 5-FU, daunorubicin was also tested in the current study. Our study indicated that daunorubicin had a very narrow therapeutic window (ocular therapeutic index of 1.25) which was also reported by the other investigators [28,29]. Although perioperative daunorubicin perfusion with vitrectomy had shown marginal efficacy in a clinical study [4], therapeutic effect of a simple ocular application without a delivery system may be very limited. In contrast, HDP-PMEG had a high ocular therapeutic index and was rationally designed for intravitreal long lasting and slow release. HDP-PMEG may be a better therapeutic than 5-FU and daunorubicin for intraocular application to manage unwanted ocular proliferation. HDP-PMEG, like 5-FU, is a metabolite which interferes with DNA synthesis and cell proliferation. It is not surprising that all three tested compounds caused a certain degree of cytotoxicity in the Müller cell line, even at a low concentration, because the tested cells were perpetually proliferating cells. In an in vivo situation, most retinal cells are quiescent cells with minimal replication. Studies have shown that a 1 mg 5-FU intravitreal injection into rabbit eye (5 mM) caused no significant inhibition of protein synthesis in photoreceptor cells and ganglion cells [30]. In a human study, a 200 µg/ml (1.53 mM) infusion during a vitrectomy seemed to be safe [5,31]. The safety and toxicity of HDP-PMEG for intraocular use needs to be investigated in vivo studies.

It has been suggested that the genotoxicity of PMEG is comparable to that of mitomycin C [9] which is used more often than 5-FU as an adjuvant for glaucoma filtering surgery [32]. The current study showed that HDP-PMEG possesses a similar genotoxicity to 5-FU. The benefit of an effective short-term treatment such as intraocular infusion during vitrectomy is clear. In this way, a drug may be given as a single intraoperative dose and washed away following the treatment period, thus controlling the therapeutic exposure more precisely.

In the current study, we also investigated HDP-PMEG on three melanoma cell lines and the results were encouraging. HDP-PMEG demonstrated an equivalent cell proliferation-arresting effect on SP6.5 and M23 cell lines to 5-FU and improved potency on OCM1 cells. HDP-PMEG is a hydrophobic crystalline compound and becomes a suspension in water-based solutions. The suspension may be directly injected into the tumor mass without being quickly distributed systemically to cause systemic side effects.

In summary, the current study evaluated HDP-PMEG in vitro for its antiproliferative potency and cytotoxicity while both 5-FU and daunorubicin were used as a comparison. Ocular cell lines were used throughout the study. Cell lines have been extensively used in vitro for drug screening to determine the inhibitory growth activity of drugs [33]. Usage of well developed cell lines facilitates the comparison among compounds and studies. We acknowledge that primary cultured cells may differ from cell lines. However, recently, two studies demonstrated that primary cultured RPE and ARPE19 responded similarly to the tested toxicants [34,35]. This may be due to the similar gene expression related to apoptosis [36], through which drug or toxicants exert their growth inhibition or cytotoxicity. The in vitro data are encouraging and suggests that HDP-PMEG may be useful in managing unwanted ocular proliferation such as various PVR and retinal surface proliferation. However, its efficacy and safety need to be studied further in small experimental animal models.
